# The role of dipole interactions in hyperthermia heating colloidal clusters of densely-packed superparamagnetic nanoparticles

**DOI:** 10.1038/s41598-018-23225-5

**Published:** 2018-03-16

**Authors:** Rong Fu, Yuying Yan, Clive Roberts, Zeyu Liu, Yiyi Chen

**Affiliations:** 1 Centre for Fluids & Thermal Engineering Research, University of Nottingham Ningbo China, Ningbo, 315100 China; 2 Fluids & Thermal Engineering Research Group, Faculty of Engineering, University of Nottingham, Nottingham, NG7 2RD UK; 3 School of Pharmacy, University of Nottingham, Nottingham, NG7 2RD UK

## Abstract

This work aims to investigate the influence of inter-particle dipole interactions on hyperthermia heating colloidal clusters of densely-packed Fe_3_O_4_ nanoparticles at low field intensity. Emulsion droplet solvent evaporation method was used to assemble oleic acid modified Fe_3_O_4_ particles into compact clusters which were stabilized by surfactant in water. Both experimental and simulation works were conducted to study their heating performance at different cluster’s sizes. The dipole interactions improve the heating only when the clusters are small enough to bring an enhancement in clusters’ shape anisotropy. The shape anisotropy is reduced at greater clusters’ sizes, since the shapes of the clusters become more and more spherical. Consequently, the dipole interactions change to impair the heating efficiency at larger sizes. When the clusters are totally isotropic in shape, the heating efficiency is lower than that of non-interacting particles despite the cluster’s size, although the efficiency increases by a little bit at a particular size most likely due to the dipole couplings. In these situations, one has to use particles with higher magnetic anisotropy and/or saturation magnetization to improve the heating.

## Introduction

Lots of efforts have been made to study magnetic hyperthermia due to its potential to be a cancer treatment^1–6^. The theory of this treatment is proposed based on the fact that magnetic nanoparticles (MNPs) convert magnetic energy to heat under an AC magnetic field *via* a mechanism called magnetic losses. Technically, a hyperthermia treatment can be delivered to a tumor as long as the particles locate within or stay close to the tumor and provide a heating strong enough to rise up the temperature of surrounding tissue by 5–10 °C. A variety of approaches have been developed to achieve tumor-targeted accumulation of MNPs, including arterial injection of MNPs into the supply vessel of tumor^4^, direct injection^7–11^, or targeted particle delivery^12–15^. One of the biggest challenges is how to obtain an efficient heating at low field intensity and frequency. Experiments show that the product of field amplitude and frequency has to be lower than 5 × 10^9^ Am^−1^s^−1^ to conduct a safe treatment.^16^ Moreover, to our knowledge, the commercialized clinical induction heating system is able to provide an oscillating field with a frequency of 100 kHz and field strength variable in the range of 2.5–15 kA/m^17^.

At lower field intensity, small MNPs are supposed to produce more heat than big ones do, since triggering the magnetic losses of large particles requires a threshold field amplitude stronger than their coercivity field^18,19^. When the sizes of MNPs are reduced to below a critical value (about 20 nm), the moments of the particles will flip freely through Néel relaxation above blocking temperature, thus coercivity field will become negligible. These particles are referred to as superparamagnetic nanoparticles (SMNPs). Such a small size leads to a very short relaxation time. According to the theory of Rosensweig^20^, higher field frequency is demanded to maximize specific absorption rate (SAR) of SMNPs when the relaxation time is shortened. Enhancing magnetic anisotropy of particles turns out to be a good option to improve the heating performance of SMNPs. Besides selecting SMNP with stronger individual magnetic anisotropy, it has been indicated that inter-particle dipole interactions can enhance the effective magnetic anisotropy^21^ by generating the dipolar magnetic field to help delaying the relaxation of the particle moments.

Colloidal clusters composed of multi MNPs have shown great potential in hyperthermia heating^22–24^. It has been reported that the dipole interactions can improve the heating when the clusters are highly anisotropic in shape, such as in chain or cylinder^23,25,26^. Mehdaoui *et al*.^23^ demonstrated that dipole interactions improved the heating performance of SMNP columns by generating an additional magnetic uniaxial anisotropy, which is favored by lower particles’ individual anisotropy and longer column. Branquinho *et al*.^27^ suggested that the optimal individual anisotropy should shift to the lower value when dipole interactions increase the effective magnetic anisotropy of chain clusters and the optimal value of individual anisotropy increases with damping factor. However, to assemble nanoparticles into clusters of high shape anisotropy, there should be anisotropic elements incorporated within the synthesis procedure, such as strong magnetic attractions among particles^28,29^ or using anisotropic building block^30^ and/or anisotropic template^31^. Moreover, the improvement benefits from reducing the angle between the field direction and the axis of the chain or cylinder^25,26,32^. It could be hard to get the clusters aligned with the field direction in tumor tissue due to the great viscosity of the tissue. A worse heating will be gained when they happen to be perpendicular to each other^32^.

To the contrary, it is much easier to synthesize anisotropy-less clusters of densely-packed SMNPs. Emulsion droplet evaporation method has been proven to be successful in massively preparation of almost mono-dispersed sphere-like clusters of densely-packed Fe_3_O_4_ nanoparticles^33^. There are only a few works carried out to study heating behaviour of clusters of densely-packed SMNPs. Different opinions were presented. Hayashi *et al*.^22^ observed that SAR was improved by 60% after assembling 9 nm of Fe_3_O_4_ nanoparticle into sphere-like clusters with size of 100 nm. Liu *et al*.^34^ observed a reduction of SAR after densely loading 6 nm of SMNPs into a sphere-like polymer latex, but using 18 nm of SMNPs brought an SAR improvement. The results of Dutz *et al*.^35^ suggested that the SAR of sphere-like clusters of iron oxide nanoparticles changed in a non-monotonical way with increasing the hydrodynamic diameter of the clusters and an optimal size for hyperthermia heating existed. Guibert *et al*.^36^ observed that formation of large and dense aggregates would result in a significant decrease in SAR. In the last year, Casula *et al*.^24^ obtained very efficient hyperthermia heating with using sphere-like Mn-doped iron oxide clusters of 45 nm. The clusters provided 100 W per gram of metal of SAR when the product of field amplitude and frequency was only 2 × 10^9^ Am^−1^s^−1^.

The present work investigated the effect of dipole interactions on the hyperthermia heating colloidal clusters of densely packed Fe_3_O_4_ nanoparticles at different cluster’s sizes. Emulsion droplet solvent evaporation method was used to assemble oleic acid (OA) modified Fe_3_O_4_ particles into the clusters. The sizes of the clusters were characterized with DLS measurements. Five sizes were obtained ranging from 70–130 nm. SAR was measured at each size under low field intensity and frequency. A standard Monte Carlo approach was implemented to perform timely dependent magnetization of the clusters of densely packed particles. Both experiment and simulated results showed that the role of dipole interactions was heavily dependent on the size of the cluster. Dipole interactions can improve hyperthermia heating only when the clusters are small enough to bring an enhancement in clusters’ shape anisotropy. If the clusters are totally isotropic in shape, it could be hard to obtain an efficient heating unless increasing individual magnetic anisotropy and/or saturation magnetization.

## Results and Discussions

### Clusters of Fe3O4 nanoparticles

**Figure 1:**
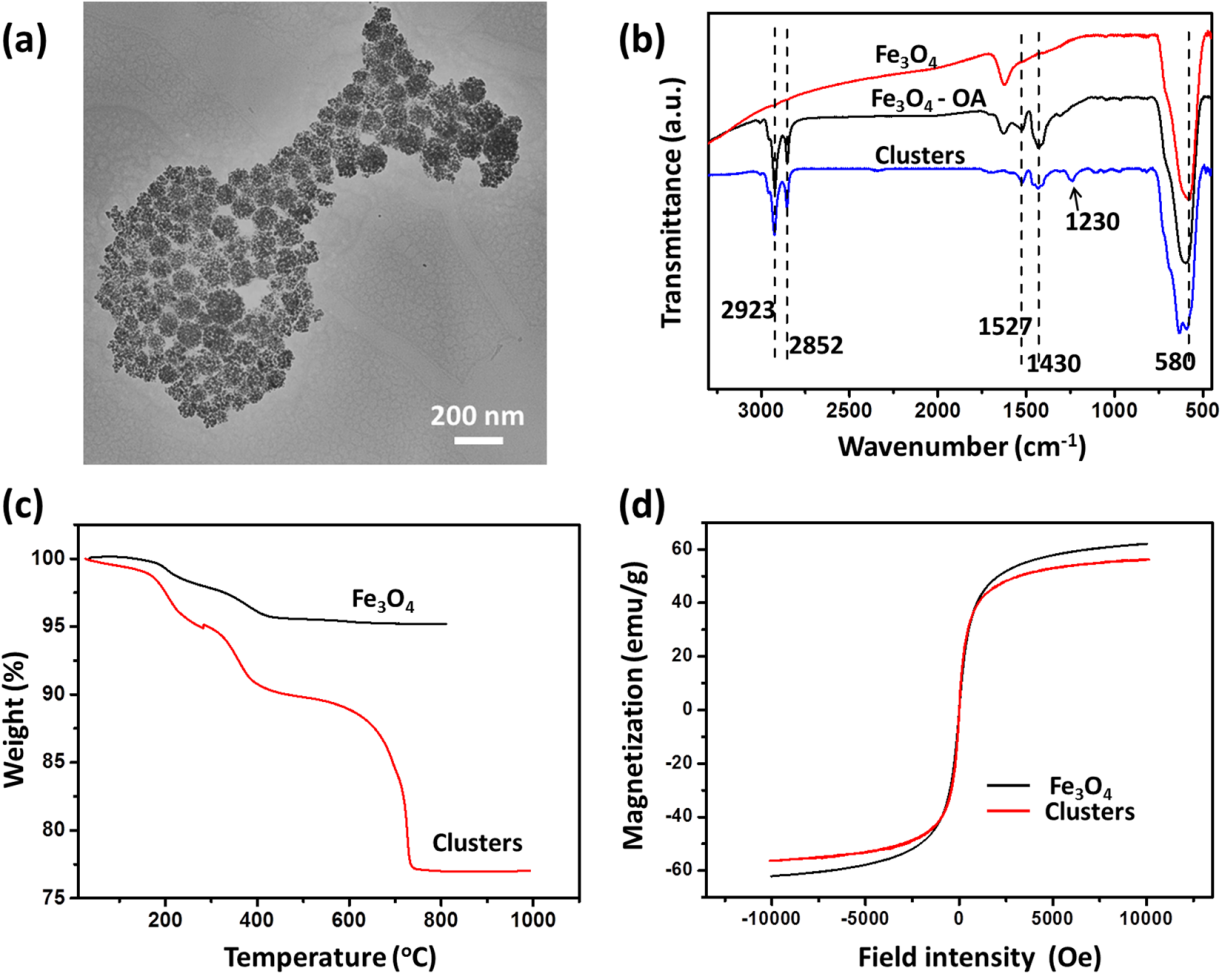
(**a**) TEM image of the clusters of Fe_3_O_4_ nanoparticles numbered by c4. (**b**) FTIR spectrums of OA modified and unmodified Fe_3_O_4_ nanoparticles and the clusters. (**c**) TGA measurements and (**d**) DC magnetizations of unmodified Fe_3_O_4_ nanoparticles and the clusters at room temperature.

Colloidal clusters were produced by assembling Fe_3_O_4_ nanoparticles via emulsion droplet solvent evaporation method. First, Fe_3_O_4_ nanoparticles prepared by the Massart method were modified with OA and dispersed in cyclohexane to form an oil phase. Then, the oil phase was mixed with a water solution of surfactant by sonication to obtain a mini-emulsion. After evaporation of the cyclohexane, due to the solvophobic interaction between the OA layer and water phase^37^, the hydrophobic Fe_3_O_4_ nanoparticles formed compact aggregates in the solution. Table 1 give the recipe for preparing the clusters of different sizes. A representative TEM image of the clusters numbered by C4 is shown in Fig. 1a. Each cluster consisted of multi Fe_3_O_4_ particles. It was found that the clusters numbered by C1 and C2 underwent severe aggregations on the TEM grid (see Supplementary Information Figure S1) most likely due to surfactant desorption. Thus, DLS measurements were conducted to determine the cluster’s sizes. As shown in Table 1, by changing the dosages of particle and surfactant, clusters of fives sizes ranging from 70–130 nm were gained. The polydispersion indexes, PdI are all below 0.2, suggesting relatively narrowed size distribution.

**Table 1:** Synthesis recipe of Fe_3_O_4_ clusters of different sizes.

	Particles (mg)	V_oil_ (ml)	SDS (mg)	V_water_ (ml)	D_H_ (nm)	PdI
c1	60	1.5	375	30	73	0.161
c2	60	1.5	150	30	94	0.186
c3	120	1.5	75	30	114	0.179
c4	240	1.5	75	30	122	0.111
c5	360	1.5	75	30	134	0.096

D_H_ is the intensity-weighted mean hydrodynamic diameter.

PdI is the polydispersion index supplied by the equipment.

V_oil_ is the volume of cyclohexane.

FT-IR characterization demonstrated that the OA were still located on the surface of the Fe_3_O_4_ nanoparticles after the assembling. As shown in Fig. 1b, the strong vibrations at 580 cm^−1^ observed on all spectrums are attributed to Fe-O stretching of Fe_3_O_4_ nanoparticles^38–40^. Compared with unmodified Fe_3_O_4_ nanoparticles, both the modified particles and the clusters present strong peaks at 2923 and 2852 cm^−1^, attributed to the vibration of CH_2_, and 1527 and 1430 cm^−1^, caused by the stretching of COO-Fe, suggesting the presence of OA on the particle surfaces^41–43^. The peak at 1230 cm^−1^ is caused by S = O stretching of the residual surfactant^44^.

TGA analysis was used to determine the mass fraction of iron oxide of the clusters (Fig. 1c). The weight loss before 500 °C was associated with removals of surface-adsorbed organic solvent and physically absorbed OA, and, perhaps, the decomposition of amorphous iron hydroxides^45^ since Fe_3_O_4_ particles also lost weight in this range. Given that Fe_3_O_4_ particles did not lose weight above 500 °C, the weight loss starting at 600 °C can be attributed to the removal of the OA directly bonded to the particles^46^. The mass faction of iron oxide of the clusters was found to be 77%.

Figure 1d shows the DC magnetizations of unmodified Fe_3_O_4_ nanoparticles and the clusters at room temperature. Both curves present negligible hysteresis loops, suggesting that they are all superparamagnetic. Due to the modification of OA, the saturation magnetization of the clusters is lower than that of unmodified particles. XRD analysis shows that the crystal structure of Fe_3_O_4_ nanoparticle maintained despite the modification of OA and the latter assembling (see Supplementary Figure S2).

### Experiments of hyperthermia heating

**Figure 2:**
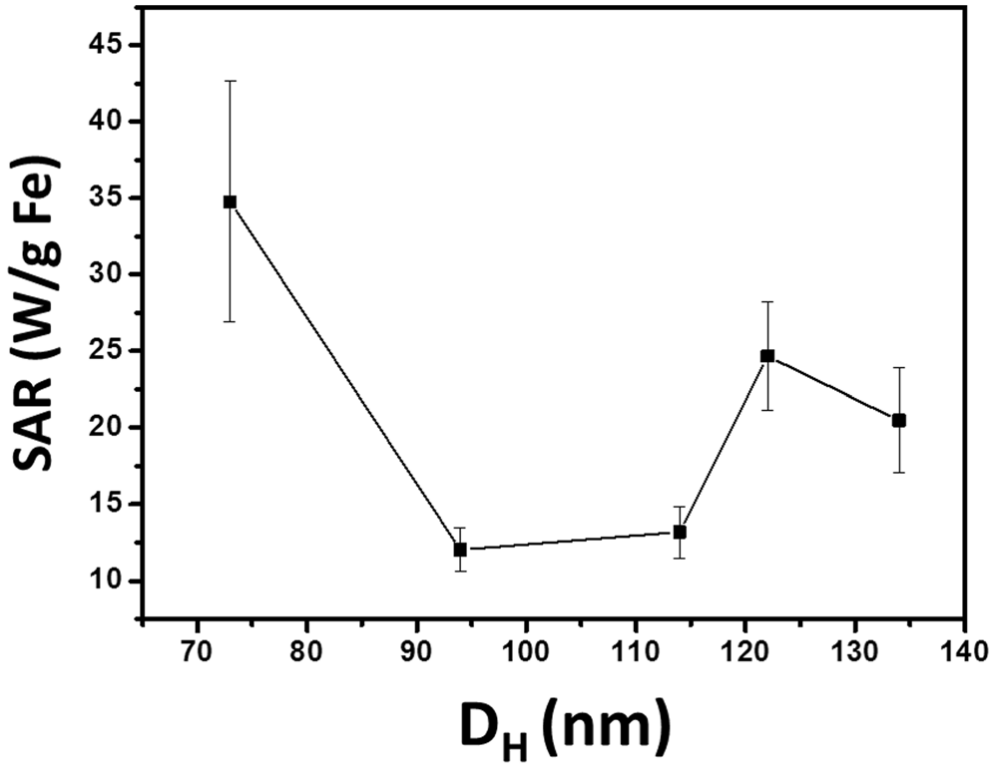
Experimentally obtained SAR of the colloidal clusters of densely packed Fe_3_O_4_ nanoparticle against D_H_. The errors are determined by the standard deviation of measuring results. The average error is 15%, indicating that the measurements were relatively precise.

Figure 2 shows SAR of the colloidal clusters of densely packed Fe_3_O_4_ nanoparticles at different cluster’s sizes. The field frequency and amplitude was 80 kHz and 13.1 kA/m respectively. At the first glance, as increasing D_H_, SAR decreases sharply at first and then changes to increase; after achieving a maxima, SAR decreases again. The location of the peak corresponds to the clusters numbered by C4, whose real size was found to be 90 nm through TEM image analysis. Compared with the result reported by Dutz *et al*.^35^, there is one more decrease of SAR occurred before the peak appears. This circumstance is similar to the finding of Ovejero *et al*.^47^ who studied the influence of particle concentration on SAR of liquid suspension of 20 nm of iron oxide particles. They suggested that the hysteresis loop is dominated by the values of maximum magnetization M_max_ and remaining magnetization. Conde-Leboran *et al*.^48^ also reported a similar picture about the relationship between particle concentration and heating performance of an ensemble of ferromagnetic particles. They interpreted the peak located at larger concentration as a transition from a major to minor loop, which was also related with the variance of M_max_.

### Simulation analysis

To investigate the role of dipole interactions, simulation work based on standard Monte Carlo approach was implemented to perform timely dependent magnetization of clusters of densely packed SMNPs. At the beginning, a bunch of mono-sized particles are numerically assembled into compact clusters. With fixing the number of the particles, the average size of the clusters is controlled by the number of the clusters. See details in Method Section. All the clusters are simultaneously subjected to the same oscillating field. Temperature is set at 298 K. The easy axes of the particles are randomly chosen in 3D space.

In one Monte Carlo step (MCS), the moment of each particle is agitated within a spherical segment around the particle’s present direction with an aperture angle θ. θ_0_ at certain temperature can be calculated according to the reported work^49^. The magnitude of θ is related to the time scale of MCS. Nowak *et al*.^50^ pioneered the work on relating one MCS to real time scale, finding that the time scale of one MCS increases with increasing the size of the aperture. Russell and Unruh *et al*.^51^ confirmed that the coercivity of non-interacting MNPs decreased and eventually disappeared as increasing the aperture angle when temperature was above 0 K and the number of MCSs per field cycle was fixed. In this work, a parameter *a* is added to change the magnitude of θ, so θ = *a* θ_0_. It was also found that when the number of MCSs per field cycle is fixed, hysteresis loop area of non-interacting particles, A_non_ decreases to zero with increasing *a* (see Supplementary Figure S3). Therefore, in fact, larger *a* leads to longer time scale of one MCS, and consequently the particles are given with more time to relax the magnetization. In other words, the increase of *a* corresponds to lowering down the field frequency.

**Figure 3:**
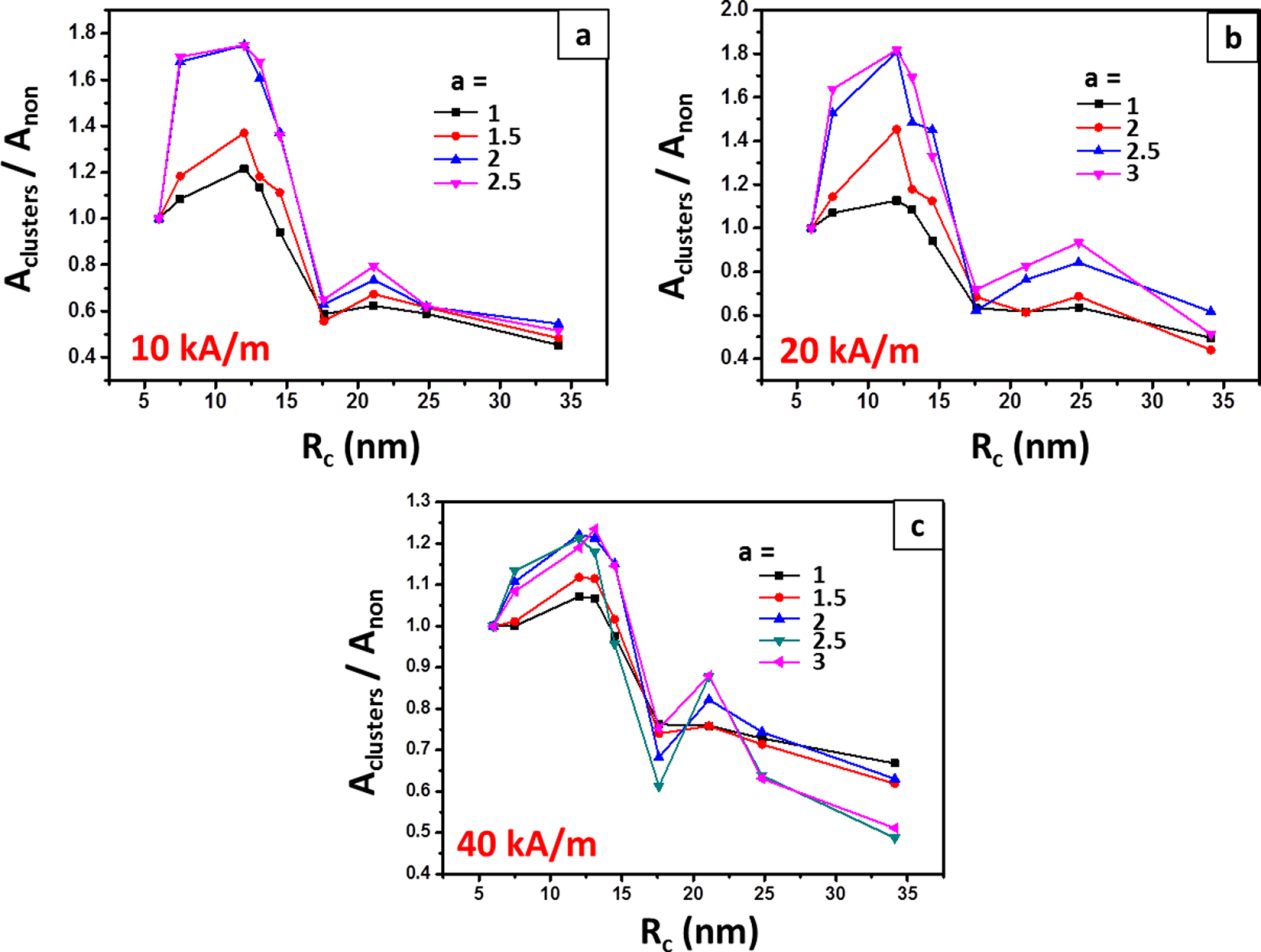
The ratio of hysteresis loop area of the clusters to that of non-interacting particles as a function of the R_c_. Data are extracted from the Monte Carlo simulations of Fe_3_O_4_ clusters subjected to an AC magnetic field.

Figure 3 gives the whole picture about the effect of clusters’ sizes on hyperthermia heating at the field amplitude of 10, 20 and 40 kA/m. The hysteresis loop area of the clusters is denoted by A_cluster_. The average radius, R_c_ is used to characterize the sizes of the clusters. The ratio of A_cluster_ to A_non_ is applied to show the influence of dipole interactions on the magnetic losses of the clusters. The first point located at 6 nm in each chart represents the result of non-interacting particles. At the first glance, there should be two peaks: the first one, peak p1 appears when the radius of cluster is around R_c_ of 12–13 nm; the second one, peak p2 can be found at R_c_ of 20–25 nm. The experimental result shown in Fig. 2 should only give the latter part of peak p1 and the peak p2. The failure in predicting the location of peak p2 may be caused by the mismatches between the properties of real particles and those used in simulations. The peak p1 is higher than 1, suggesting improved magnetic hysteresis at R_c_ of 12–13 nm. In all three cases, the peak p1 grows higher with increasing *a* and finally reaches the maximum height. The whole peak p2 is lower than 1, indicating that a worse heating could be expected at this region.

**Figure 4:**
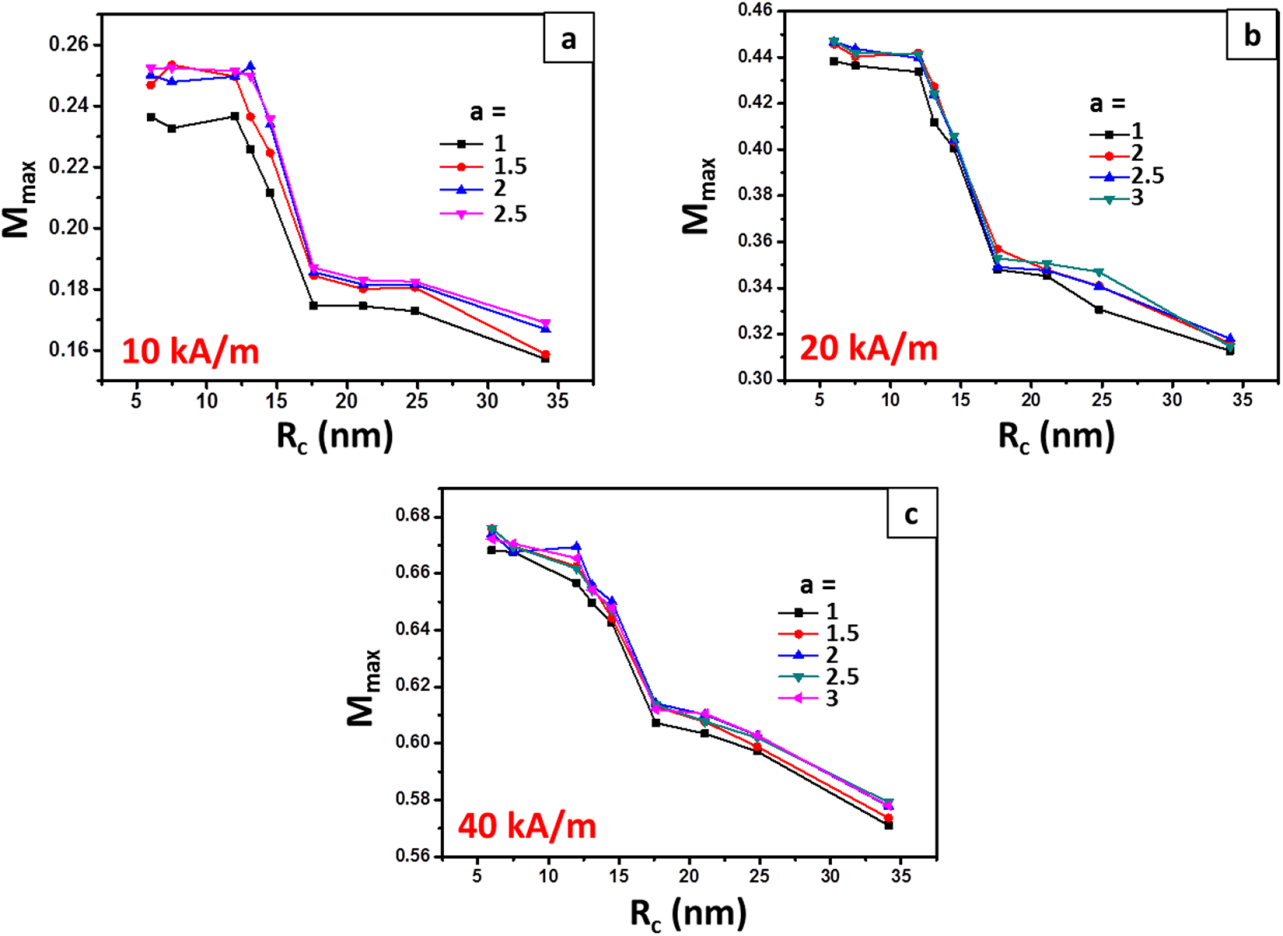
Maximum magnetization of the cluster normalized by saturation magnetization as a function of the R_c_. Data are extracted from the Monte Carlo simulations.

Figure 4 shows M_max_ normalized by saturation magnetization M_s_ as a function of the R_c_. The major loop, mentioned in the work of Conde-Leboran *et al*.^48^, represents the kind of magnetic losses in which a particle system achieves the M_s_. It can be seen that all normalized M_max_ shown in Fig. 4 are lower than 1, despite the field amplitude and *a*. Thus, the occurrences of both two peaks have little to do with the transition from a major to minor loop. It also can be seen that after experiencing a short plateau first, M_max_ decreases sharply as the radius increases from 12.5 to 17.5 nm. With further increasing the R_c_, there is no peak locating on the profile of M_max_. M_max_ still keeps decreasing but at lower rate. Therefore, the occurrences of both the peaks are not related with the variance of M_max_ too.

**Figure 5:**
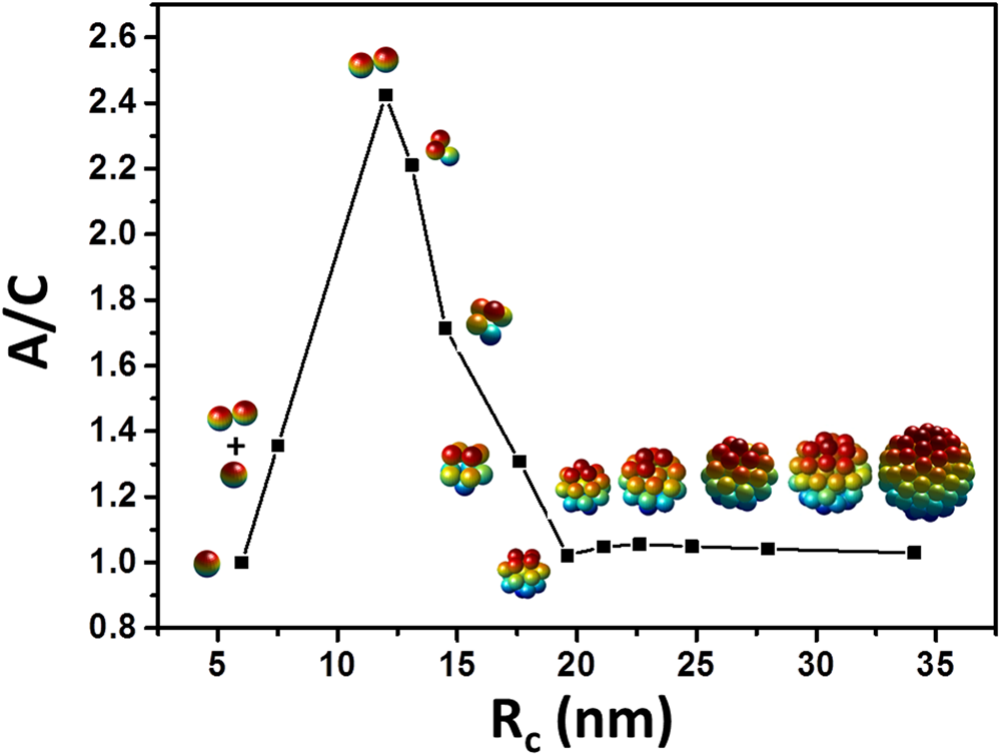
The change of the shape anisotropy as a function of the R_c_ of the numerically fabricated clusters.

It has been suggested that anisotropic shape can bring improved heating performance. Hence, the shape anisotropy of numerically fabricated clusters is characterized. The clusters are treated as equivalent ellipsoids of uniform mass distribution in 3D. The shape anisotropy is defined in terms of the ratio of the longest semi-principal axis, *A* to the shortest one, *C* of the ellipsoid. The ratio is averaged over all the clusters at each R_c_. Detailed instruction is supplied in our previous publication^32^. Figure 5 shows the shape anisotropy as a function of R_c_ and 3D schematics of the typical cluster at each R_c_. The first point at 6 nm represents isolated particle. The largest ratio of *A* to *C* is 2.5 and obtained when the radius of cluster is 12 nm, which is the diameter of single particle plus the thickness of organic layer. At this size, the shape of each cluster is a dimer containing two particles. Increasing the clusters’ sizes from 12 to 18 nm leads to a reduction in shape anisotropy, since the shapes of the clusters are becoming more and more like spheres. The variance of shape anisotropy is consistent with the change of A_cluster_ in the region of peak p1. So, the occurrence of peak p1 must be related to the improved shape anisotropy.

**Figure 6:**
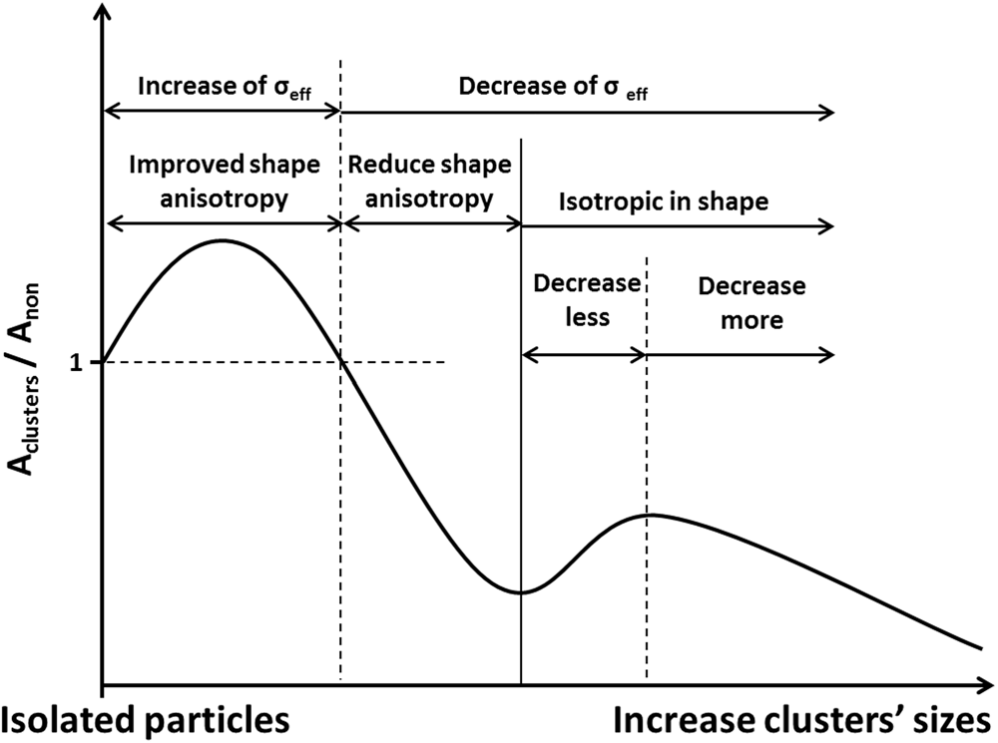
Schematic description of the magnetic hyperthermia scenario of densely packed clusters.

Here, we come up with a general scenario describing the role of dipole interactions in hyperthermia heating the clusters of densely packed SMNPs. It is schematically illustrated in Fig. 6. Branquinho *et al*.^27^ suggested that the effect of dipole interactions on magnetic losses of SMNPs can be interpreted as the influence on the effective magnetic anisotropy, ${\sigma }_{{eff}}$, a dimensionless anisotropy term which is normalized by thermal energy. When the cluster is anisotropic in shape, dipole interactions improve the heating by generating a new magnetic anisotropy^23^, as a result, ${\sigma }_{{eff}}$ is improved. And it should be favoured by lowering down the frequency, given that the peak p1 grows higher with *a* (see Fig. 3).

However, once the clusters are losing shape anisotropy, the improvement brought by this new magnetic anisotropy will be reduced. Moreover, when dipole interactions are not so strong that the energy barrier for moment’s spin is still mainly determined by individual anisotropy, enhancing dipole interactions will change to reduce the effective magnetic anisotropy^52^. This explains why peak p1 appears at the region of smaller size. When the clusters become totally isotropic in shape, the reduced ${\sigma }_{{eff}}$ impairs the heating efficiency. The occurrence of peak p2 is most likely due to the dipole couplings, which should hamper relaxation of moments caused by thermal flocculation and brings an influence similar to retarding the reduction of ${\sigma }_{{eff}}$. At lower field frequency, particles are supposed to be given with more time to form stronger dipole couplings. This explains why a sharper peak p2 is obtained at larger *a* (see Fig. 3). However, after further increasing the cluster’s size, too strong dipole interactions accelerate the decrease of ${\sigma }_{{eff}}$, leading to a more worse heating efficiency.

**Figure 7:**
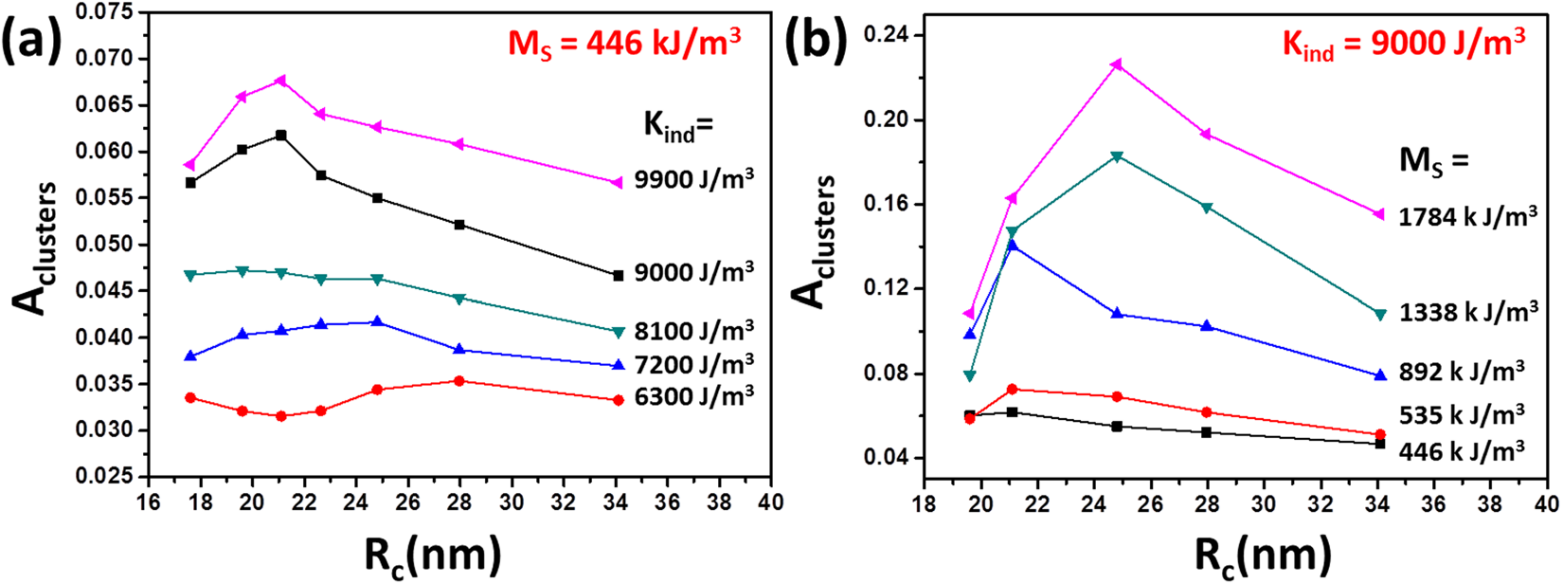
Simulation – determined hysteresis loop area of the clusters isotropic in shape as a function of the R_c_ at different individual magnetic anisotropy (**a**) and saturation magnetization (**b**).

From the discussion above, it can be conclusive that it is difficult for clusters isotropic on shape to heat better than the non-interacting particles do. However, it is important to understand how to enhance their heating efficiency. After all, there are several works which reported efficient heating with using sphere-like clusters^22,24,34^. Figure. 7 shows the A_cluster_ of the clusters isotropic in shape at different individual anisotropy ${K}_{{ind}}$ and M_s_. The field amplitude is kept at 40 kA/m and *a* equals 2. The peak p2 is flattened after intentionally decreasing the ${K}_{{ind}}$ (Fig. 7a). A reduced ${K}_{{ind}}$ should facilitate the moments’ relaxation, thus it becomes more difficult to form dipole couplings. And the loop area decreases with lowering ${K}_{{ind}}$ despite the R_c_. It proves that without the shape anisotropy, the energy barrier for moment’s spin is determined by individual anisotropy. The peak p2 grows higher as increasing M_S_ from 446 to 1784 kJ/m^3^ (Fig. 7b). The optimum R_c_ is still in the range of 20–25 nm. An increased M_S_ reinforces not only dipole couplings but also the coupling with the external field. Therefore, if one expects compact clusters without shape anisotropy to heat more efficiently, SMNPs with larger M_S_ and ${K}_{{ind}}$ must be used.

In short, based on the analysis of experimental and simulated results, a general picture was presented to describe the role of inter-particle dipole interactions in hyperthermia heating the clusters of densely-packed SMNPs as a function of cluster’s size at low field intensity. Dipole interactions are inclined to improve the hyperthermia heating only when the clusters are small enough to induce an enhancement in clusters’ shape anisotropy. Once the clusters are losing their shape anisotropy, dipole interactions will change to impair the heating. When the clusters are totally isotropic in shape, it is hard for them to provide a heating better than non-interacting particles do, even though the heating efficiency could rebound by somewhat at a particular size. To make them heat more efficiently, SMNPs with stronger saturation magnetization and individual magnetic anisotropy must be used.

## Methods

### Reagents

Ferric chloride (FeCl_3_ · 6H_2_O, ≥ 99%), ferrous chloride (FeCl_2_ · 4H_2_O, ≥ 99%), ammonium hydroxide (25%), oleic acid (OA, 90%), sodium dodecylsulfate (SDS, 99%), cyclohexane (99.8%), ethanol (200 proof), hydrochloric acid (HCl, 37%) were all purchased from Sigma Aldrich. De-ionized water was used for preparing all aqueous solutions.

### Preparation of clusters of densely packed Fe_3_O_4_ nanoparticles

Fe_3_O_4_ nanoparticles were synthesized by co-precipitation method^33,53^. Briefly, FeCl_3_ · 6H_2_O and FeCl_2_ · 4H_2_O was dissolved in water according to molar ratio of 2:1. Then, ammonium hydroxide was added under vigorous agitation. After 30 min, black precipitate of Fe_3_O_4_ nanoparticles of average size of 10 nm was collected at the bottom of flask.

In the following, the surfaces of the Fe_3_O_4_ particles were modified with OA. 0.008 mol of Fe_3_O_4_ particles were dispersed to 100 mL of water with the aid of sonication. The pH of the particle suspension was adjusted to 8.5 by adding diluted HCl solution. The dark suspension was heated to 70 °C and kept stirred under the protection of N_2_. 1.33 mL of oleic acid was added to the suspension. After 2–3 h, all of particles precipitated down to the bottom of the flask. The oily precipitates were washed with ethanol for 5 times and then dispersed to cyclohexane to form a magnetic oil phase.

Emulsion droplet solvent evaporation method was used to assemble OA modified Fe_3_O_4_ particles into colloidal clusters of densely packed particles^33^. Table 1 give the recipe for preparing the clusters of different sizes. In a typical procedure, 1.5 mL of the magnetic oil phase was mixed with 30 mL of aqueous SDS solution with aid of sonication to form a mini-emulsion system. The system was stirred at 60 °C for 5–6 h under the protection of N_2_ to evaporate cyclohexane. At last, a centrifugation was used to separate the colloidal clusters from the solution. After dumping the supernatant, the clusters were re-dispersed to a diluted SDS solution. The volume of the diluted SDS solution was adjusted so that the final concentration of the cluster was 40 mg / mL.

### Characterizations

Transmission electron microscopy (TEM) images were observed under a JEOL-2000 electron microscope operating at 200 kV. X-Ray powder diffraction (XRD) pattern was obtained with a Bruker D8 Advance Powder X-ray diffractometer. Thermogravimetric analysis (TGA) was carried out on a TGA-SDTQ600 thermogravimetric analyser. The sample was heated to 1000 °C under the protection of N2. Fourier transform infrared (FT-IR) spectra were gained with a Perkin-Elmer Spectrum One FT-IR spectrophotometer. All FT-IR samples were prepared into KBr tablets, and the number of scans was set at 20 to collect the spectra. Before TGA and FT-IR measurements, the colloidal clusters were washed with water to remove the absorbed SDS. A Malvern Zen 3600 Zetasizer was applied for dynamic light scattering (DLS) measurements. All the samples for DLS measurements were diluted first to 0.002 vol % with diluted SDS solution. A JDM-13 vibrating sample magnetometer was used to characterize the superparamagnetism of the Fe3O4 nanoparticles and the clusters. The field-dependent magnetization was analyzed over a range from −10 to + 10 kOe at room temperature.

### Magnetic hyperthermia

A 80 kHz of AC magnetic field was provided by a copper solenoid connected to a Roy 1500 Induction Heater (Fluxeon Inc., USA). The field amplitude was measured to be 13.1 kA/m. All the samples were water solutions of the clusters, and the concentration was kept at 40 mg/mL. The sample was put into a glass-made container jacketed with vacuum layer, which was placed at the centre of the solenoid. During the induction heating, the solenoid was cooled by circulating water flow. Temperature of the sample was probed with a fluoro-optic fiber thermometer (OPSENS SOLUTIONS INC, Canada). The SAR is defined as the thermal power dissipation divided by the mass of metal content, which is given as,

$${\bf{S}}{\bf{A}}{\bf{R}}=\frac{{{\boldsymbol{C}}}_{{\boldsymbol{p}}}{\boldsymbol{\rho }}{{\boldsymbol{V}}}_{{\boldsymbol{s}}}}{{\boldsymbol{m}}}\frac{{\boldsymbol{dT}}}{{\boldsymbol{dt}}}$$

where C_p_ is the specific heat capacity, ρ the density, V_s_ the volume, and m the mass of metal content of the sample, dT/dt is the initial linear rise in temperature versus time dependence. Since the concentration of the sample is quite low, the C_p_ and ρ were set as the same as those of water, which is 4.186 J/(g K) and 1.0 g/mL. The measurement was taken 5 times for each sample.

### Simulation

It is assumed that all the particles are mono-sized and ideally spherical in shape. The radius of the particle is set at 5 nm. The thickness of organic layer is typically assumed to be 1 nm. 100 of such particles are distributed randomly to N_g_ of groups. The number of the particles in each group should not be lower than the integer part of 100/N_g_. To numerically assemble the particles of each group into a compact cluster, the particles are let to move freely in a large sphere and take ideally elastic collisions when they meet. The collision between a particle and the wall of the sphere is also treated as elastic collision. Meanwhile, the radius of this sphere decreases slowly until it cannot be reduced any more. This is judged by that the reduction in the radius at each time becomes negligible (less than 10^−6^ nm). The final radius is used as the radius of this cluster. The radius is averaged after the particles of all the groups are assembled into clusters. The averaged radius R_c_ is used to characterize the sizes of the clusters. Reducing the N_g_ increases the R_c_.

A standard Monte Carlo approach featured with the Metropolis algorithm was carried out to perform timely dependent magnetization. Each particle possesses a uniaxial magnetic anisotropy, and the orientation of the easy axis is randomly chosen in 3D space. The magnetic properties of the particle are set the same as the published data of magnetite nanoparticles: the individual anisotropy constant K_ind_ is 9000 J/m^3^ and saturation magnetization M_s_ 446 kA/m^54^. For the sake of simplicity, it is just assumed that both of them are temperature-independent. Temperature is 298 K. The energy model consists of three major sources, namely, anisotropy energy, Zeeman energy and dipolar interaction energy. A detailed description of the model and simulation processing was supplied in our previous publication^32^. At each R_c_, all the clusters were simultaneously subjected to the same AC magnetic field. The magnetization of the system is collected by summing the moment projections on the positive direction of field variation. The hysteresis loops are computed for typically 200 times for averaging purpose.

## Electronic supplementary material

Supplementary Information
