# Supplementary material for: The role of dipole interactions in hyperthermia heating colloidal clusters of densely-packed superparamagnetic nanoparticles
Source: Sci Rep. 2018 Mar 16;8:4704. doi: 10.1038/s41598-018-23225-5 (PMC5856762; doi:10.1038/s41598-018-23225-5)
Supplement: Supplementary file 1 — Supplementary Information [file 41598_2018_23225_MOESM1_ESM.pdf]

The role of dipole interactions in hyperthermia heating of colloidal clusters of densely-packed superparamagnetic nanoparticles

Rong Fu[1], Yuying Yan[1,2*], Clive Roberts[3], Zeyu Liu[2], Yiyi Chen[2]

1. Centre for Fluids & Thermal Engineering Research, University of Nottingham Ningbo China, Ningbo, 315100, China

2. Fluids & Thermal Engineering Research Group, Faculty of Engineering, University of Nottingham, Nottingham, NG7 2RD, UK

3. School of Pharmacy, University of Nottingham, Nottingham, NG7 2RD, UK

Corresponding author: Yuying Yan (Yuying.yan@nottingham.ac.uk)

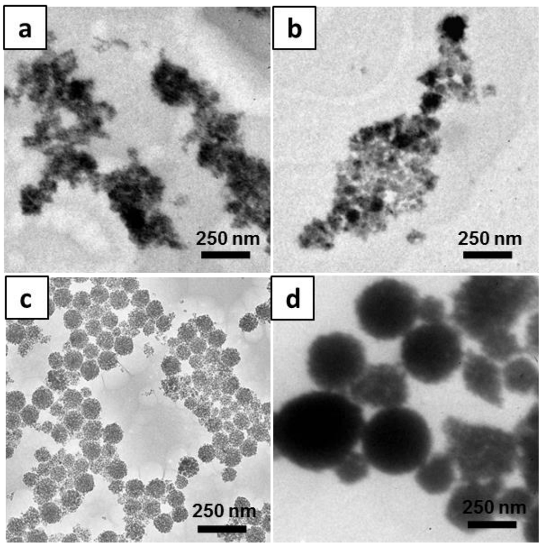

Figure S1. TEM images of the clusters of densely packed Fe_3O_4 nanoparticles numbered by c1 (a), c2 (b), c3 (c) and c5 (d). It was identical that the cluster numbered by c1 and c2 underwent severe aggregations on the TEM grid. Given that the surface of the grid was hydrophobic, the aggregations were most likely due to desorption of surfactant absorbed on the surfaces of the clusters. Therefore, DLS measurements were conducted to characterize the sizes of the clusters.

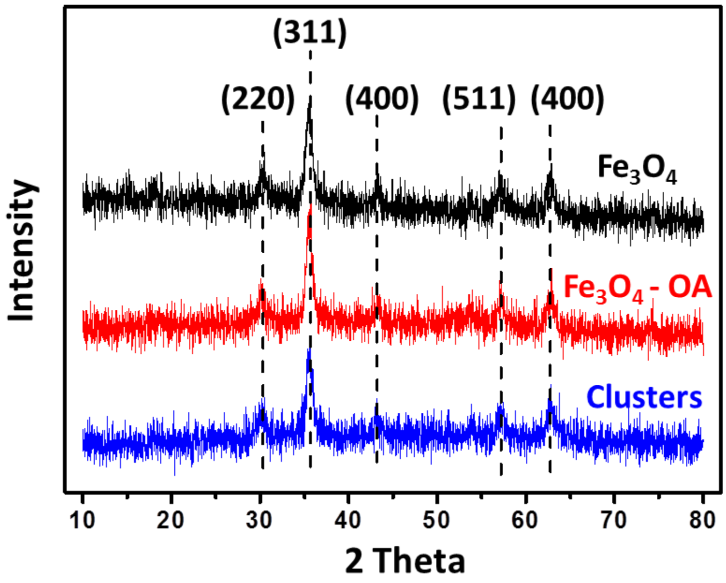

Figure S2. XRD patterns of oleic acid modified and unmodified Fe_3O_4 nanoparticles and the clusters of densely packed Fe_3O_4 nanoparticles. It was found that the patterns of the modified particles and the clusters both presented the same characteristic peaks of cubic inverse spinel structure as those of the unmodified Fe_3O_4 nanoparticles. Therefore, there was no change in crystal structure of the Fe_3O_4 nanoparticles after the modification of oleic acid and latter assembling.

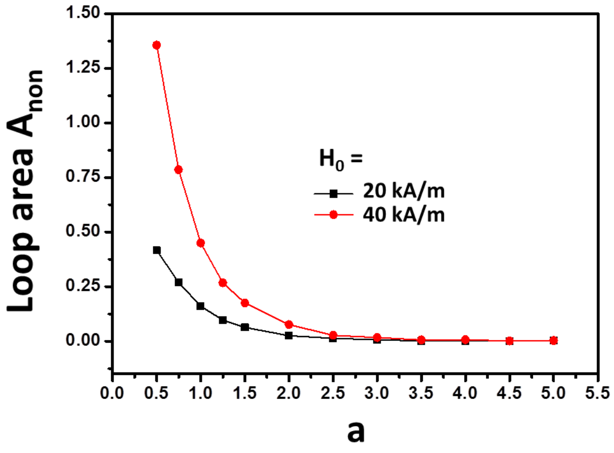

Figure. S3 Simulated hysteresis loop area of non-interacting particles, A_{non} as a function of *a*. In the main text, it has been suggested that the magnitude of the aperture angle is related with the real time scale of one Monte Carlo step. The value of the aperture angle θ_0 at certain temperature can be calculated according to the reported work (see Ref 49). In this work, a parameter *a* is added to change the magnitude of θ, so $\theta = a\ \theta_0$. It can be seen that A_{non} decreases to 0 as increasing *a*. This suggests that increasing *a* leads to longer time scale of one

Monte Carlo step so that the particles are given more time to relax the magnetization. From another perspective, the increase of a affects the magnetic losses in the same way as lowering down the field frequency.
